# Supplementary material for: Uncovering mutation-specific morphogenic phenotypes and paracrine-mediated vessel dysfunction in a biomimetic vascularized mammary duct platform
Source: Nat Commun. 2020 Jul 6;11:3377. doi: 10.1038/s41467-020-17102-x (PMC7338408; doi:10.1038/s41467-020-17102-x)
Supplement: Supplementary file 2 — Supplementary Information [file 41467_2020_17102_MOESM2_ESM.pdf]

SUPPLEMENTARY INFORMATION

Uncovering mutation-specific morphogenic phenotypes and paracrine-mediated vessel dysfunction in a biomimetic vascularized mammary duct platform

Kutys, Polacheck et al.

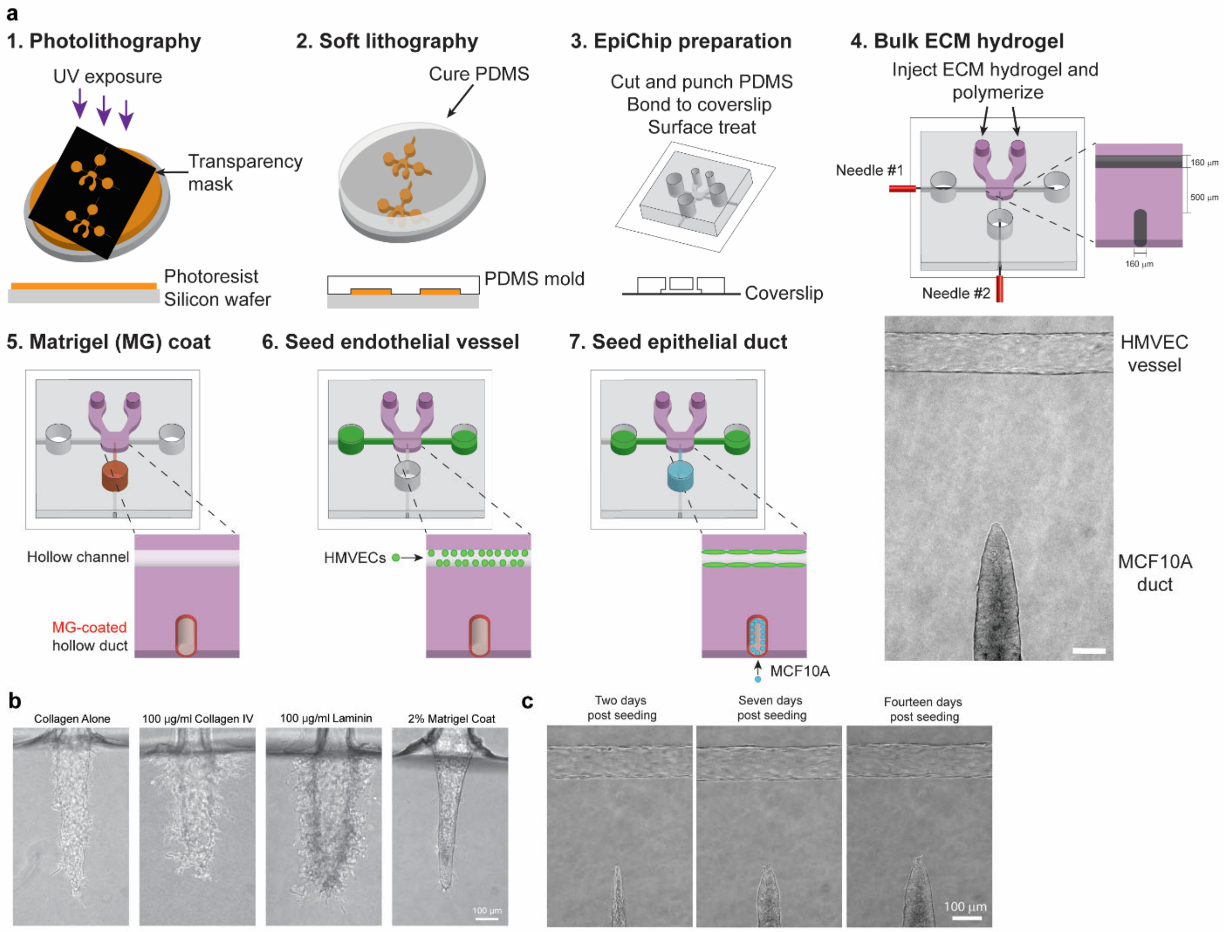

Supplementary Figure 1: (a) Overview of fabrication process for the co-culture platform. 1) Three-layer photolithography is required to make the silicon master mold. 2) PDMS is cured on the master mold to make microfluidic devices. 3) The PDMS devices are cut, bonded to glass, and surface treated to allow the hydrogel to stick to the PDMS surface inside the device. 4) Two steel needles are introduced into the device prior to injecting a 3D hydrogel (e.g. collagen, fibrin) as a liquid and polymerizing the gel around the needles. 5) The steel needles are removed to create a hollow central channel and a perpendicular blunted channel that is then passively coated with dilute growth factor reduced Matrigel (GFR MG). 6) Primary hMVEC endothelial cells are introduced into the central channel in solution and allowed to adhere to the inside surface of the channel. 7) After allowing cells to adhere and spread, MCF10A mammary epithelial cells are introduced to the blunted channel in solution and allowed to adhere to the MG-coated surface. Endothelial vessels are maintained under perfusion by pump or rocking platform and the epithelium matures over five days for stable co-culture (phase contrast image of each tissue within a single device, scale bar 100 µm). (b) Phase contrast images of the representative morphology of MCF10A ducts three days after seeding into duct cavities with no ECM coating (collagen alone) or coating with collagen IV (100 µg/ml), laminin (100 µg/ml), or 2% Matrigel (~160 µg/ml). (c) Phase contrast images of a MCF10A/hMVEC co-culture device at two, seven, and fourteen days post seeding. All images are representative of at least three independent experiments.

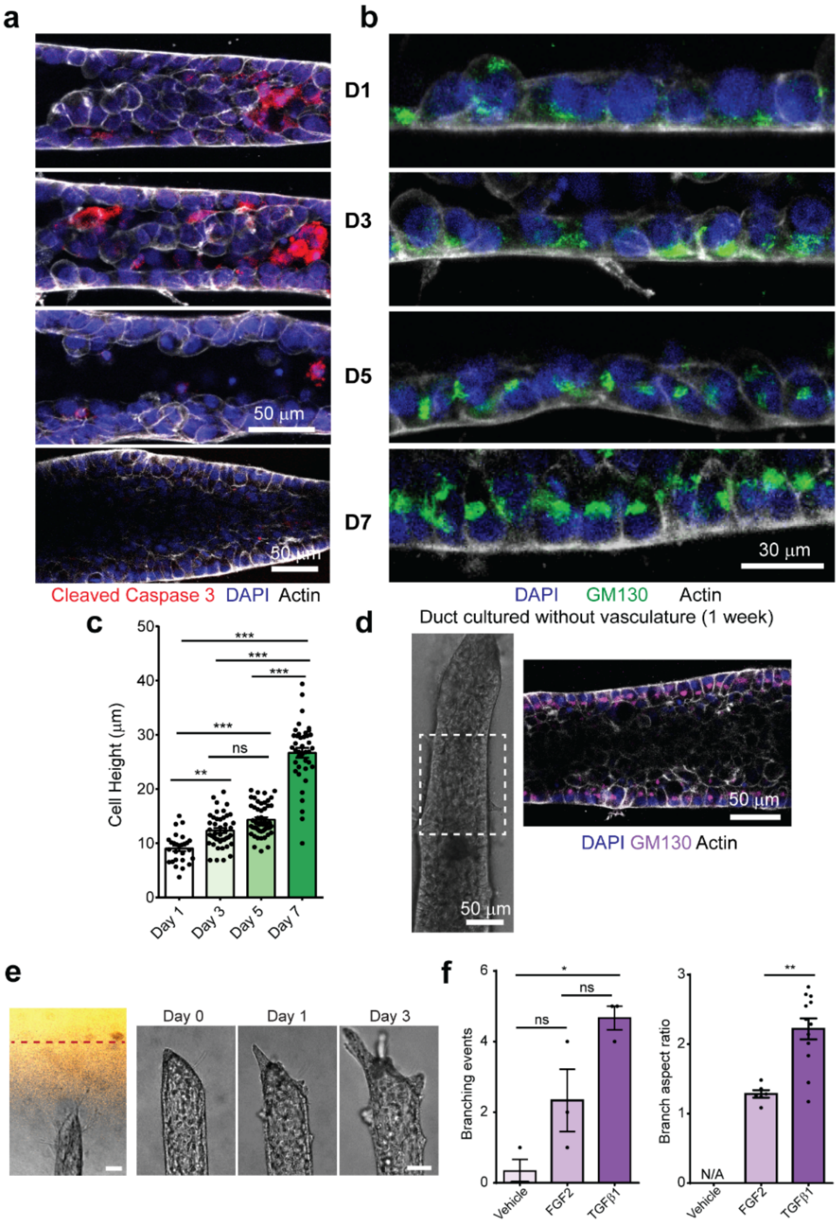

Supplementary Figure 2: Micrographs of confocal sections of ducts over the time course of duct stabilization immunostained for (a) cleaved caspase 3 (red) or (b) GM130 (green) along with actin (white) and nuclei (blue). (c) Quantification of mammary epithelial cell height (defined as length from basal collagen interface to top of the cell) during the time course of duct stabilization (n=27,44,53,40 cells from across three independent duct experiments one-way Anova with Bonferroni post-test, Day 1 vs Day 3 **P=0.0032, Day 1 vs Day 5 ***P= 1.61229e-08, Day 1 vs Day 7 ***P= 5.03066e-42, Day 3 vs Day 7 ***P= 1.49506e-38). (d) Phase contrast image and corresponding confocal slice micrograph of a mammary duct at one week in culture without a vascular compartment. (e) Linear morphogen gradients established by diffusion from the acellular vascular channel after one hour (orange, 70 kDa FITC-dextran). Phase contrast time course of TGFβ1 gradient associated morphogenesis of mammary ducts (scale bars 50 µm). (f) Quantification of branching events and branch aspect ratio for FGF2 and TGFβ1 treated ducts (n=3,3,3 ducts from three independent experiments; ; one-way Anova with Bonferroni post-test *P=0.0044), (n=5,12 branched regions from three independent ducts experiments; two-tailed, unpaired Student's *t*-test, *P=0.0006). For all plots, values mean ± s.e.m. and all images are representative of at least three independent experiments.

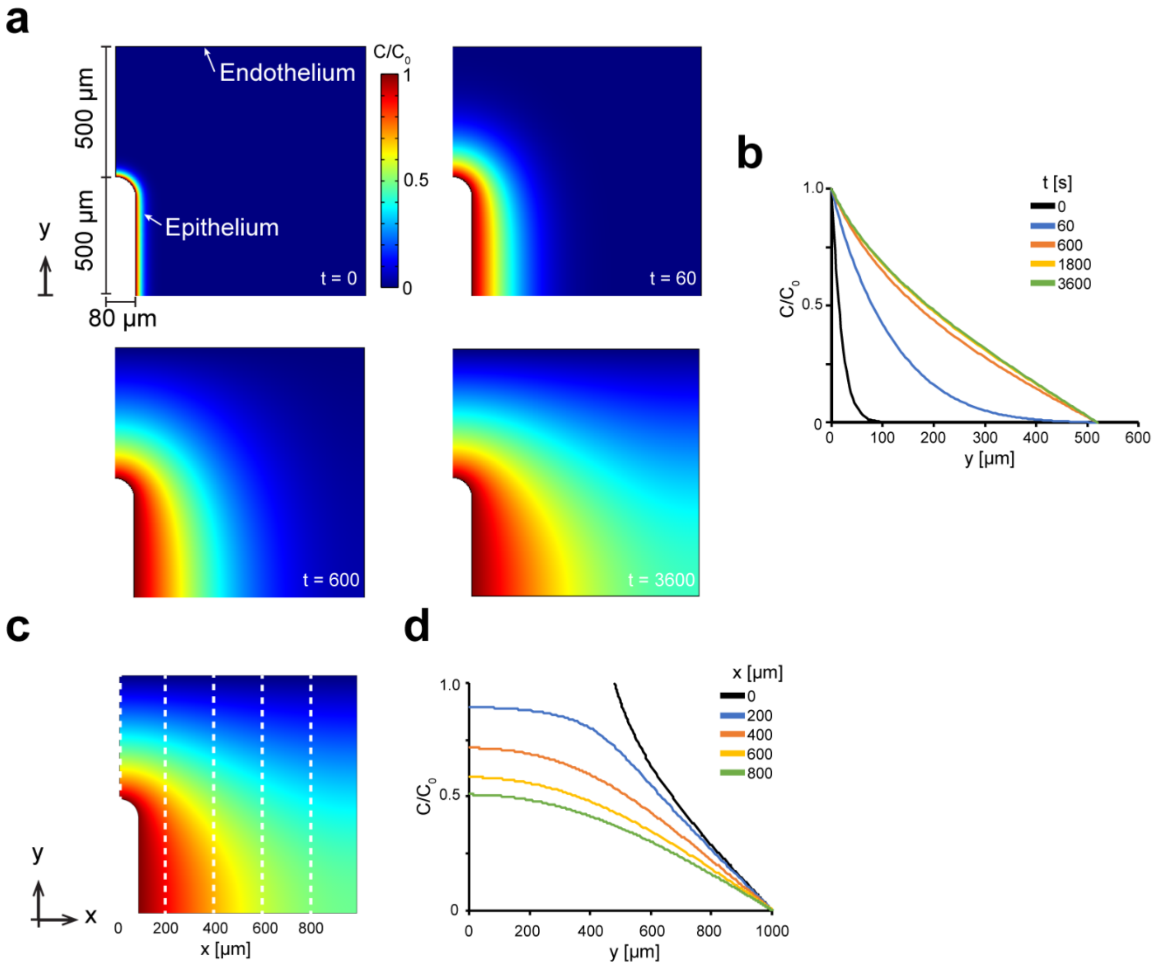

Supplementary Figure 3. Finite element simulation of transport in microfluidic platform. (a) Heatmap of the concentration of cytokine released from the epithelial channel over time (in seconds). (b) Quantification of molecular concentration at the tip of the epithelial duct demonstrates that a gradient is established within hours. (c) Concentration profile at steady state and (d) plotting concentration profiles normal to the endothelial channel at increasing distance from the epithelial channel demonstrates that molecular gradients are established at the endothelial wall.

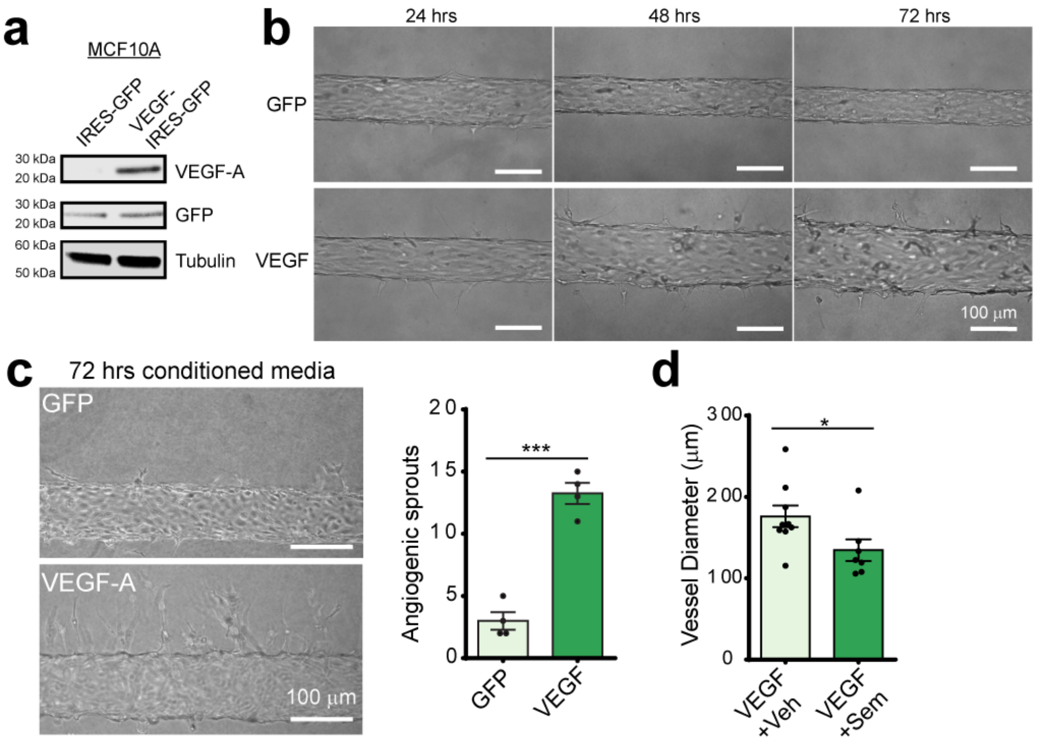

Supplementary Figure 4: (a) Western blot of IRES-GFP and VEGF-IRES-GFP expressing MCF10A lysates. (b) Phase contrast time course of endothelial vessels co-cultured with GFP or VEGF-expressing ducts. (c) Phase contrast images of endothelial vessels treated for three days with GFP or VEGF-expressing conditioned medium. Quantification of multicellular angiogenic sprouts in response to conditioned media (n=4,4 vessels examined across three independent experiments; two-tailed, unpaired Student's *t*-test ***P= 9.04588e-5). (d) Quantification of the diameter of vessels in co-culture with VEGF-expressing ducts and treated with DMSO (Vehicle) or 10 μM Semaxanib (n=9,7 vessels examined across three independent experiments; two-tailed, unpaired Student's *t*-test *P=0.0484). For all plots, values mean ± s.e.m. and all images are representative of at least three independent experiments.

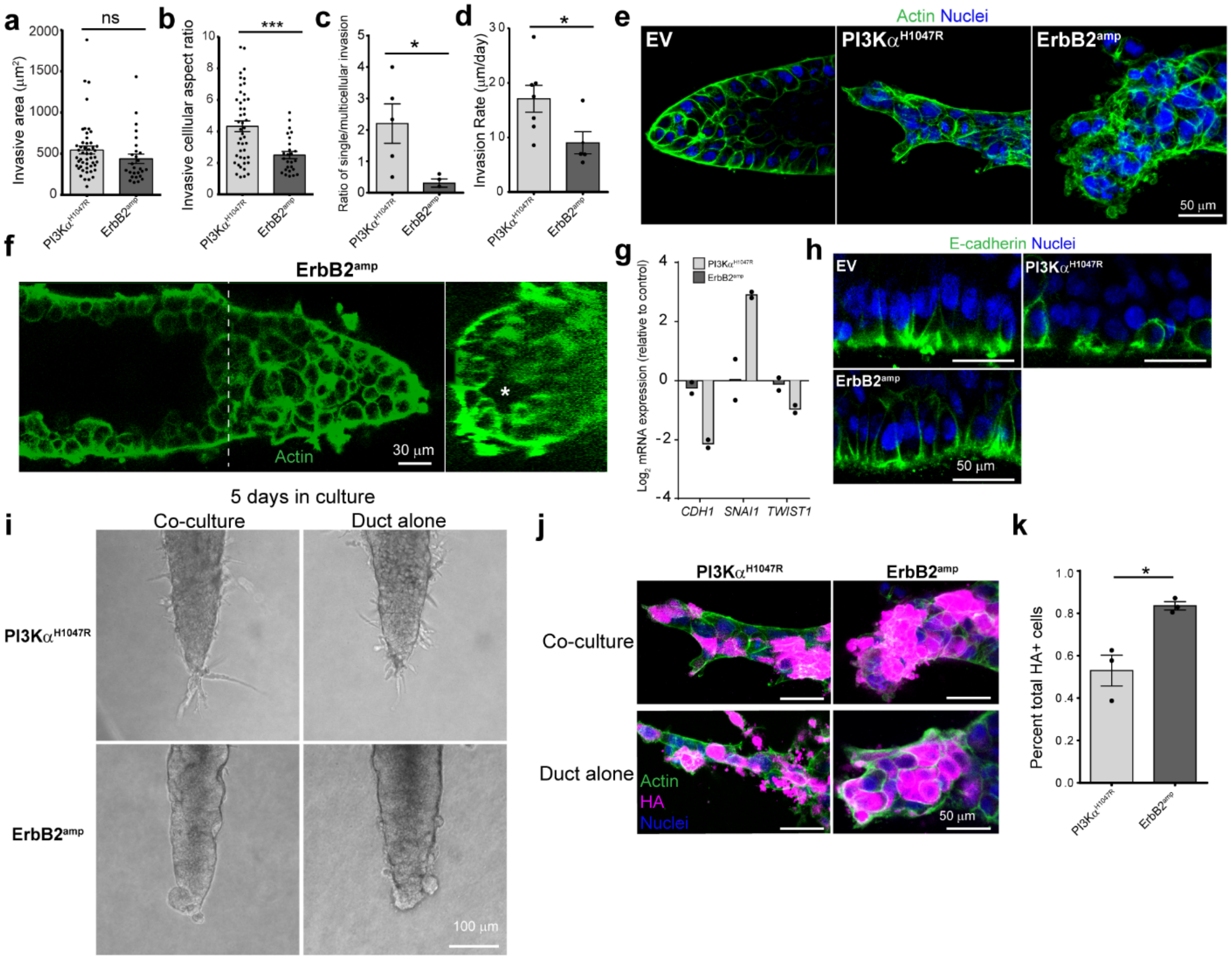

Supplementary Figure 5: Quantification of (a) invasive area from the initial duct geometry (n=49,27 invasive processes from ten independent ducts; two-tailed, unpaired Student's *t*-test, P=0.1699), (b) cellular aspect ratio (ratio of long to short axis) of cells within the invasive front (n=49,27 cells examined from three independent duct experiments; two-tailed, unpaired Student's *t*-test, ***P=0.0003), (c) ratio of individual versus collective migration in each invasive front (n=5,4 ducts examined across three independent experiments; two-tailed, unpaired Student's *t*-test, *P=0.0339), and (d) invasion rate of each migration front, for PI3Kα^{H1047R} and ErbB2^{amp} ducts after one week in co-culture (n=7,5 ducts examined across three independent experiments; two-tailed, unpaired Student's *t*-test, *P=0.0389). (e) Maximum intensity projection micrographs of duct termini (green actin, blue nuclei). (f) Confocal slice and 3D cross-section of an ErbB2^{amp} duct. Asterisk indicates acellular duct lumen (green actin). (g) mRNA expression levels for indicated genes from PI3Kα^{H1047R} or ErbB2^{amp} cells (n=2 independent experiments). (h) Confocal slice micrographs of ducts immunostained for E-cadherin (green) and nuclei (blue). (i) Representative phase contrast images of PI3Kα^{H1047R} or ErbB2^{amp} ducts cultured with endothelial cells (left) and without endothelial cells (right) (n=2). (j) Invasive fronts of PI3Kα^{H1047R} or ErbB2^{amp} ducts cultured with endothelial cells (top) and without endothelial cells (bottom) and immunostained for the hemagglutinin (HA)-tag associated with the expression of either mutant (magenta HA, green actin, blue nuclei). (k) Quantification of the percentage of HA positive cells within the invasive front (n=3,3 invasive fronts examined from three independent duct experiments; two-tailed, unpaired Student's *t*-test, *P=0.0153). For all plots, mean ± s.e.m. and all images are representative of at least three independent experiments unless otherwise stated.

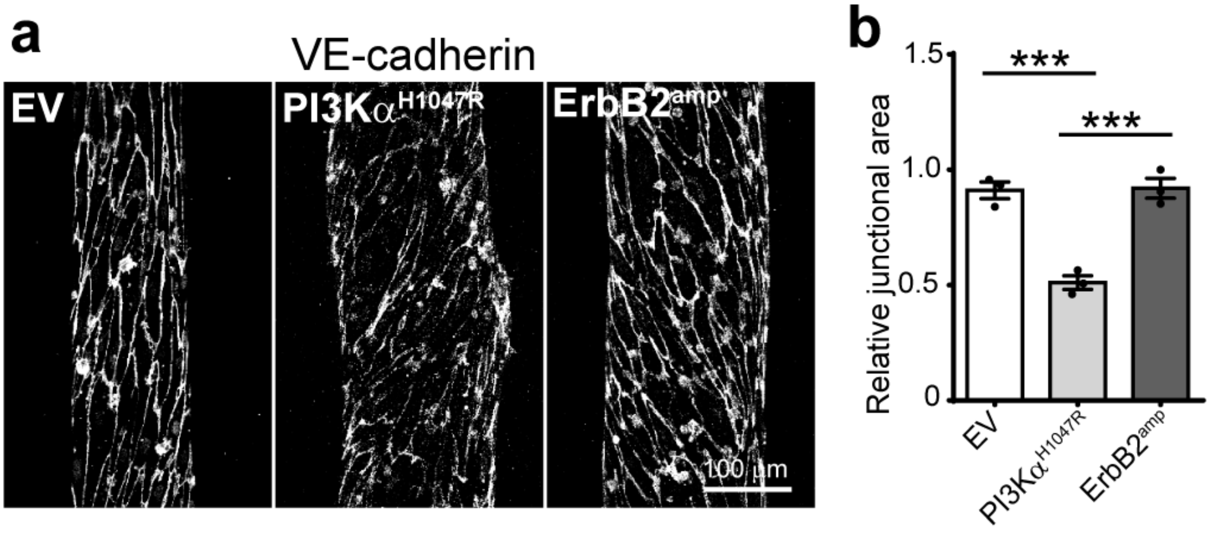

Supplementary Figure 6: (a) Maximum intensity projection micrographs of VE-cadherin in each vessel associated with the indicated duct genotype after five days in co-culture. (b) Quantification of relative junctional VE-cadherin (n=3,3,3 vessels examined across three independent experiments; one-way ANOVA with Bonferroni post-test, EV vs PI3Kα^{H1047R} ***P=0.0008, PI3Kα^{H1047R} vs ErbB2^{amp} ***P=0.0007). Plot values mean ± s.e.m. and images are representative of at least three independent experiments.

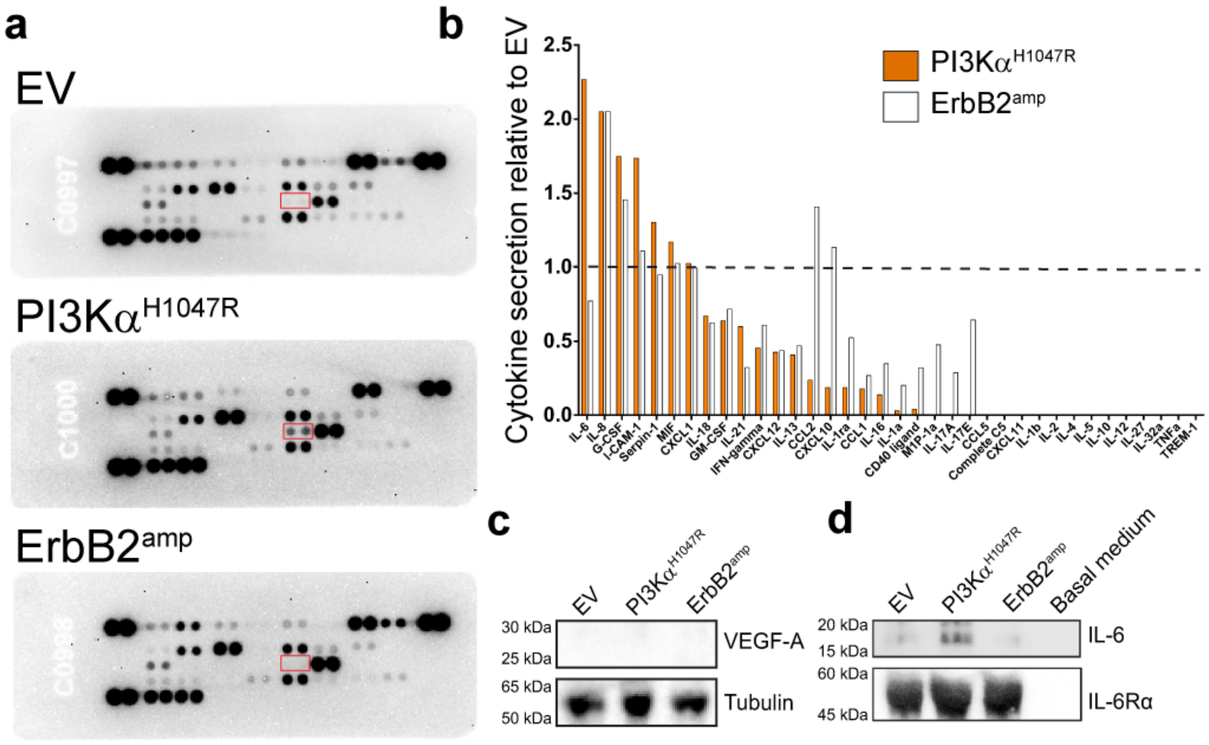

Supplementary Figure 7: (a) Results of a cytokine western blot dot array (R&D Systems, n=1) of conditioned media from indicated EV, PI3Kα^{H1047R}, and ErbB2^{amp} ducts (red box, IL-6) (b) intensity quantification of cytokine array relative to EV secretion levels (dashed line). The preliminary observation of increased IL-6 secretion with PI3Kα^{H1047R} was further validated by western blot. (c) Western blot of lysates from EV, PI3Kα^{H1047R}, and ErbB2^{amp} MCF10A. (d) Western blot of conditioned media from EV, PI3Kα^{H1047R}, and ErbB2^{amp} MCF10A and basal duct culture medium. (a,b) are the results of one experiment. Western blot images (c,d) are representative of at least three independent experiments

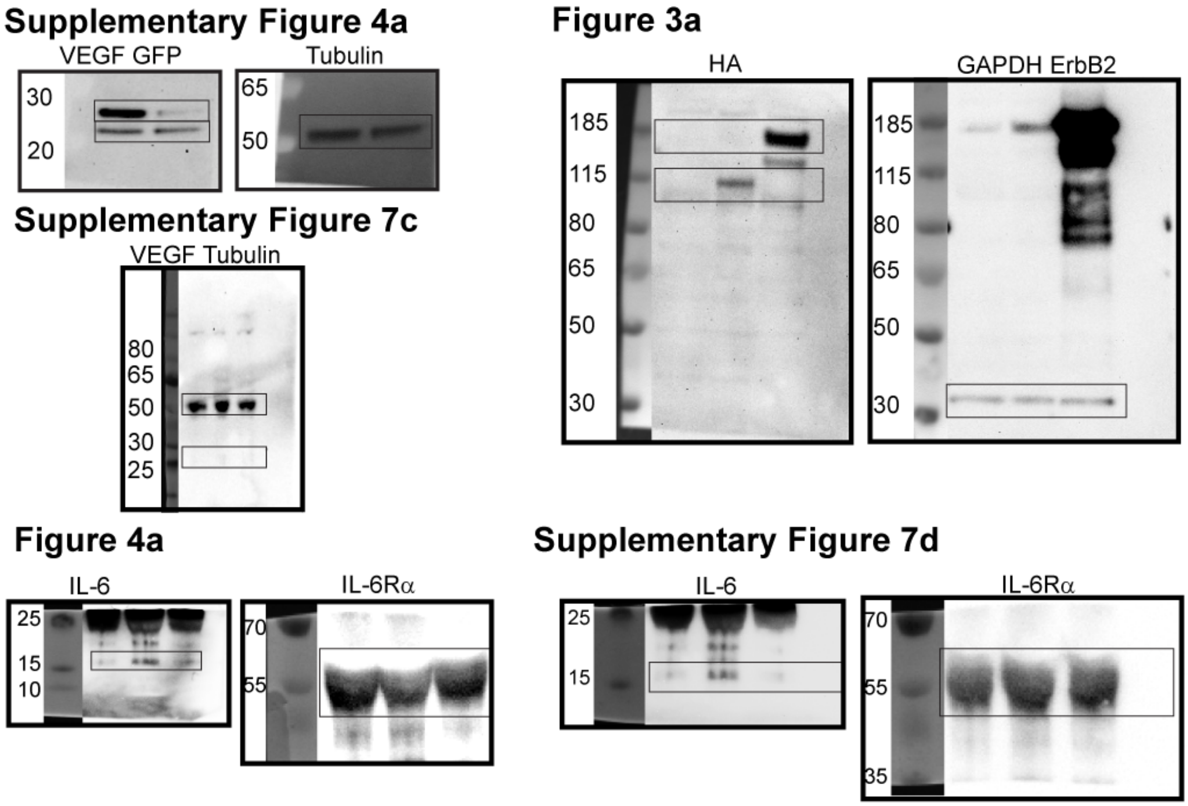

Supplementary Figure 8: Uncropped Western blot membranes for each representative image.

Supplementary Methods

Finite element model of mass transport from epithelial channel

A finite element model (FEM) was developed in Comsol Multiphysics (COMSOL, Burlington, MA) to determine the dynamics of solute transport between the epithelial and endothelial compartments. Midplane confocal slices of a typical experiment were imported into Autocad (Autodesk, San Rafael, CA) and a 2D geometry was drawn and exported to Comsol. The transport of dilute species module was used to solve the time-dependent conservation of mass equation assuming no convection throughout the hydrogel:

$$\frac{\partial C_i}{\partial t} = D_{ij}\nabla^2 C_i \quad (1)$$

where C_i is the solute concentration, t is time, and D_{ij} is the diffusion coefficient of the solute. The diffusion coefficient was assumed to be homogenous and isotropic and defined to be 2.78×10^{-10} m^2s^{-1}, informed by experimental measurements of diffusion of VEGF[1], which has a similar molecular weight and expected diffusion coefficient as IL-6. No flux boundary conditions were applied at PDMS walls and the symmetry plane. To determine the appropriate boundary conditions at the endothelial and epithelial walls, we first consider flow through the endothelial channel as driven by the rocker. Assuming viscous-dominated flow in the channel, the relationship between flow rate and pressure drop is given by the Hagen-Poiseuille equation:

$$Q = \frac{\pi r^4}{8\mu}\left(-\frac{dP}{dz}\right) \quad (2)$$

where Q is the volumetric flow rate in, r is the channel radius, P is the fluid pressure, z is the coordinate in the direction of flow, and μ is the fluid viscosity. The pressure drop is determined by the angle of the rocker:

$$\frac{\Delta P}{l} = -\rho g \sin(\alpha) \quad (3)$$

Where, ρ is the fluid density, g is acceleration due to gravity, and α is the rocker angle. Combining Eqs. 2 and 3 and solving for the mean fluid velocity gives the relation for average flow velocity magnitude in the vessel as a function of rocker angle:

$$v = \frac{r^2}{8\mu}\rho g \sin(\alpha) \quad (4)$$

Therefore, with r = 80μm, α_{max}=25°, ρ = 10^3 kg m^{-3}, and μ = 10^{-3} Pa s, the average velocity is 3.3×10^{-3} m s^{-1}. We can thus compare the rate of transport due to diffusion vs. convection by scaling Eq. 1 to determine the Peclet number (Pe) in the endothelial channel:

$$\mathrm{Pe} = \frac{vL}{D_{ij}} \quad (5)$$

Using the average velocity computed above, the diffusion coefficient for VEGF, and the diameter of the channel, as we are interested in the relative rates of diffusion from the surrounding gel into the channel, we find Pe > 10^3. This indicates transport in the endothelial compartment is convection-dominated and justifies a constant concentration boundary condition in transport simulations of the hydrogel domain. Therefore, we assume a constant concentration of C_i = 0 at the endothelial wall, and we assume a constant concentration boundary condition at the wall of the epithelium. The model was solved at 30s

intervals over 2hrs. To determine concentration profiles at steady state, profiles across the chip were taken as a function of distance from the epithelial channel.

Supplementary References

1. Miura T, Tanaka R. In vitroVasculogenesis Models Revisited - Measurement of VEGF Diffusion in Matrigel. *Mathematical Modelling of Natural Phenomena.* 2009;4(4):118-130.
