# Supplementary material for: Uncovering mutation-specific morphogenic phenotypes and paracrine-mediated vessel dysfunction in a biomimetic vascularized mammary duct platform
Source: Nat Commun. 2020 Jul 6;11:3377. doi: 10.1038/s41467-020-17102-x (PMC7338408; doi:10.1038/s41467-020-17102-x)
Supplement: Supplementary file 4 — Reporting Summary [file 41467_2020_17102_MOESM4_ESM.pdf]

nature research

Corresponding author(s): Chen, Christopher S.

Last updated by author(s): Jun 2, 2020

Reporting Summary

Nature Research wishes to improve the reproducibility of the work that we publish. This form provides structure for consistency and transparency in reporting. For further information on Nature Research policies, see our Editorial Policies and the Editorial Policy Checklist.

Statistics

For all statistical analyses, confirm that the following items are present in the figure legend, table legend, main text, or Methods section.

n/a	Confirmed	
☐	☒	The exact sample size (n) for each experimental group/condition, given as a discrete number and unit of measurement
☐	☒	A statement on whether measurements were taken from distinct samples or whether the same sample was measured repeatedly
☐	☒	The statistical test(s) used AND whether they are one- or two-sided<br>*Only common tests should be described solely by name; describe more complex techniques in the Methods section.*
☒	☐	A description of all covariates tested
☐	☒	A description of any assumptions or corrections, such as tests of normality and adjustment for multiple comparisons
☐	☒	A full description of the statistical parameters including central tendency (e.g. means) or other basic estimates (e.g. regression coefficient) AND variation (e.g. standard deviation) or associated estimates of uncertainty (e.g. confidence intervals)
☒	☐	For null hypothesis testing, the test statistic (e.g. F, t, r) with confidence intervals, effect sizes, degrees of freedom and P value noted<br>*Give P values as exact values whenever suitable.*
☒	☐	For Bayesian analysis, information on the choice of priors and Markov chain Monte Carlo settings
☒	☐	For hierarchical and complex designs, identification of the appropriate level for tests and full reporting of outcomes
☒	☐	Estimates of effect sizes (e.g. Cohen's d, Pearson's r), indicating how they were calculated

Our web collection on statistics for biologists contains articles on many of the points above.

Software and code

Policy information about availability of computer code

Data collection	Microscopy data were acquired on microscope systems running commercially available MetaMorph 7 or LASX (no version #) software. Nanointendation was acquired using Optics11 DataViewer. Western blots were obtained on using an iBright imager.
Data analysis	Data were analyzed using MATLAB R2015b, ImageJ/FIJI, and GraphPad Prism 6. For quantification of vascular permeability, publicly disseminated code was used (Polacheck and Kutys et al. Nature 2017). Methodology for quantifying cytoskeletal phenotypes are included in the Methods.

For manuscripts utilizing custom algorithms or software that are central to the research but not yet described in published literature, software must be made available to editors and reviewers. We strongly encourage code deposition in a community repository (e.g. GitHub). See the Nature Research guidelines for submitting code & software for further information.

Data

Policy information about availability of data

All manuscripts must include a data availability statement. This statement should provide the following information, where applicable:

- Accession codes, unique identifiers, or web links for publicly available datasets
- A list of figures that have associated raw data
- A description of any restrictions on data availability

No restrictions on data availability and a statement is included in the manuscript. Raw source data are displayed as discrete data points on all graphs. Source data for all Figures is included in the Source Data table.

Field-specific reporting

Please select the one below that is the best fit for your research. If you are not sure, read the appropriate sections before making your selection.

- [x] Life sciences
- [ ] Behavioural & social sciences
- [ ] Ecological, evolutionary & environmental sciences

For a reference copy of the document with all sections, see nature.com/documents/nr-reporting-summary-flat.pdf

Life sciences study design

All studies must disclose on these points even when the disclosure is negative.

Sample size	Sample sizes of sufficient power were chosen on the basis of similar published research (Polacheck and Kutys et al. Nature, 2017) and were confirmed statistically by appropriate tests.
Data exclusions	No data were excluded.
Replication	All experimental data were repeated a minimum of three independent times. All attempts at replication were successful.
Randomization	No randomization was used; morphogenic phenotypes markedly distinct.
Blinding	No blinding was used; morphogenic phenotypes markedly distinct.

Reporting for specific materials, systems and methods

We require information from authors about some types of materials, experimental systems and methods used in many studies. Here, indicate whether each material, system or method listed is relevant to your study. If you are not sure if a list item applies to your research, read the appropriate section before selecting a response.

Materials & experimental systems

n/a	Involved in the study	
[ ]	[x]	Antibodies
[ ]	[x]	Eukaryotic cell lines
[x]	[ ]	Palaeontology and archaeology
[x]	[ ]	Animals and other organisms
[x]	[ ]	Human research participants
[x]	[ ]	Clinical data
[x]	[ ]	Dual use research of concern

Methods

n/a	Involved in the study	
[x]	[ ]	ChIP-seq
[x]	[ ]	Flow cytometry
[x]	[ ]	MRI-based neuroimaging

Antibodies

Antibodies used	All antibodies (supplier name, catalog number/clone name, concentration) are provided in the Methods section under "Antibodies and reagents" as follows:<br>Anti-VE-cadherin (F-8, 1 μg/ml) and DAPI were from Santa Cruz Biotechnology. Anti-E-cadherin (ab1416, 1 μg/ml), anti-Ki67 (ab15580, 0.25 μg/ml), and anti-β tubulin (ab6046, 0.25 μg/ml) were from Abcam. Anti-GM130 (clone 35, 2 μg/ml) was from BD Biosciences. Anti-VEGFA (VG-1, 2 μg/ml), anti-ZO-1 (40-2200, 1 μg/ml), rhodamine phalloidin (1 μg/ml), 70kDa FITC-dextran and AlexaFluor 647 conjugated goat secondary antibodies were from Life Technologies. Anti-α6 integrin (MA6, 1 μg/ml) was from Millipore. Anti-HA (6E2, 0.5 μg/ml), anti-GFP (D5.1, 0.5 μg/ml), and anti-GAPDH (D16H11, 0.25 μg/ml) were from Cell Signaling Technologies. HRP-conjugated donkey anti-mouse and rabbit IgG secondary antibodies (1: 0.25 μg/ml) were purchased from Fitzgerald. Recombinant human IL-6 protein (7270-IL, 200 ng/ml), recombinant human TGFβ1 protein (240-B, 5 ng/ml), recombinant human FGF2 protein 2 (33-FB, 3nM), anti-IL-6 (6708, 1 μg/ml), anti-IL-6Rα (MAB227, 0.5 μg/ml), and Proteome Profiler Human Cytokine Array Kit were purchased from R&D Systems. Semaxanib was purchased from Selleckchem.
Validation	All antibodies are commercially available with validation details and citations on the specific manufacturer website. In this study, primary antibodies were validated for use based on the position of the antigen in SDS-PAGE gels and by immunofluoresence.

Eukaryotic cell lines

Policy information about cell lines

Cell line source(s)	Human mammary epithelial line MCF10A (ATCC) were grown as<br>previously described. Briefly, cells were cultured in growth medium, consisting of DMEM/F12 (1:1,<br>Gibco) supplemented with 5% horse serum (Invitrogen), 20 ng/ml rhEGF (Peprotech), 0.5 mg/ml

	hydrocortisone (Sigma), 100 ng/ml cholera toxin (Sigma), 10 ug/ml insulin (Sigma) and 1% penicillin/streptomycin (Life Technologies). For co-culture experiments, MCF10A ducts were cultured in basal assay medium, consisting of phenol red-free DMEM/F12 (1:1, Gibco), 2% horse serum (Invitrogen), 5 ng/ml rhEGF (Peprotech), 0.5 ug/ml hydrocortisone (Sigma), 100 ng/ml cholera toxin (Sigma), 10 ug/ml insulin (Sigma) and 1% penicillin/streptomycin (Life Technologies). Human dermal microvascular endothelial cells (hMVEC-Ds, Lonza) were cultured in EGM2 medium (Lonza) supplemented with an MV2 bullet kit (Lonza). For co-culture in microfluidic devices, hMVECs were cultured in a reduced EGM2-MV, composed of the complete media kit with the exception of 0.5% serum and 0 ng/ml VEGF. HEK-293T cells (Clonetech) were grown in high glucose DMEM (Hyclone) supplemented with 10% fetal bovine serum (Hyclone) and 1% penicillin/streptomycin (Life Technologies). All cells were cultured at 37°C and 5% CO_2 in a humidified incubator.
Authentication	Cell lines were authenticated by Lonza and ATCC for virus testing (HIV, HBV, HCV), microbial testing (sterility, mycoplasma), cell performance testing (viability, cell count, seeding efficiency, doubling time), and contamination/differentiation testing (CD31 and podoplanin expression).
Mycoplasma contamination	Cell lines were tested and found negative for mycoplasma contamination using MycoAlert Mycoplasma Detection Kit (Lonza).
Commonly misidentified lines<br>(See ICLAC register)	Cell lines are not listed in database of commonly misidentified cell lines.
